# Parental perspectives on clinician guidance in value-laden NICU conversations: a qualitative study

**DOI:** 10.1186/s12887-026-07304-5

**Published:** 2026-07-28

**Authors:** Christine Arnold, Julian Eychmüller, Dina Hediger, Stephanie Vanessa Schmid, Sophie Jaisli, André Kidszun

**Affiliations:** 1 Division of Neonatology, Department of Pediatrics, Inselspital, Bern University Hospital, University of Bern, Friedbühlstrasse 19, Bern, 3010 Switzerland; 2 Frühchen & Neokinder Schweiz, Swiss non-profit organization of parents of preterm born children, Hinterer Schermen 29, Ittigen, 3063 Switzerland

**Keywords:** Neonatal decision-making, Value-laden conversations, Clinical guidance, Parental autonomy, Communication

## Abstract

**Background:**

Value-laden decisions about life-sustaining treatment for critically ill infants in the neonatal intensive care unit (NICU) present profound emotional and ethical challenges for parents and clinicians. In such situations, clinicians must balance providing guidance with respecting parental autonomy. We explored parental perspectives on clinician guidance and effective communication during these decisions.

**Methods:**

We conducted a secondary qualitative analysis within a randomized clinical trial using parental free-text responses to a simulated neonatal decision-making scenario. The study was carried out online between February and April 2024 in Switzerland, Germany, and Austria. Eligible participants were German-speaking parents of preterm infants with prior NICU experience, recruited through patient and parent organizations. Data were analyzed using qualitative content analysis.

**Results:**

Among 128 participants (84% female), parents emphasized the importance of clear, detailed, and evidence-based information about their child’s condition and treatment options, as well as empathetic, honest, and individualized communication. They valued sufficient time for discussion and reflection, access to second opinions, and attention to the child’s quality of life. Parents appeared to retain a sense of decision ownership, while differing in their preferences regarding clinician guidance. Some welcomed explicit recommendations, whereas others preferred neutral, value-sensitive communication that preserved space for independent deliberation.

**Conclusions:**

Parents facing complex medical decisions value both high-quality information and empathetic and honest communication. While they generally retain decision ownership, preferences regarding clinician guidance vary, suggesting that clinicians should actively explore how much direction parents want and tailor their communication accordingly.

**Trial registration:**

ISRCTN registry under number ISRCTN16123073, registration date: 17/01/2024, (https://www.isrctn.com/ISRCTN16123073).

## Background

Making value-laden decisions regarding treatment options for critically ill infants in the neonatal intensive care unit (NICU) presents profound emotional and ethical challenges for both parents and healthcare professionals [1–3]. These situations are often characterized by prognostic uncertainty and involve weighing complex treatment alternatives, such as whether or not to perform a tracheostomy, each associated with distinct benefits, burdens, and long-term implications [4]. Notable differences in perceptions, priorities, and emotional framing between caregivers and healthcare professionals highlight potential disconnects and underscore the need for effective communication [5]. The consequences of these decisions may be far-reaching and enduring for both the infant and the family. In this context, conversations between clinicians and parents play a central role. Decision-making requires careful consideration of personal values, ethical principles, and a balance between the interests of the infant and those of the family. Parents are frequently overwhelmed by the weight of these choices and may seek guidance from healthcare professionals. At the same time, clinicians may feel uncertain about how best to provide such guidance, as they strive to support parents while respecting their autonomy and avoiding undue influence [6, 7]. Many parents wish to be actively involved or to share in the decision-making process [8].

Central to these conversations is a trusting physician-parent relationship, which forms the foundation of high-quality care [9, 10]. A supportive and respectful relationship can empower parents to engage in complex decision-making and to act in the best interests of their child. When communication is empathetic, transparent, and collaborative, it may strengthen parents’ confidence and their ability to navigate these challenging situations [10, 11].

However, less is known about how parents perceive clinician guidance within value-laden NICU conversations, particularly whether and how they want clinicians to share recommendations or personal perspectives. It also remains unclear how parents understand their own role in these situations, including the extent to which they wish to retain decision ownership while receiving support from clinicians. Addressing these questions is essential for tailoring communication to individual needs and improving shared decision-making in neonatal care.

The aim of this study was to explore parents’ perspectives on clinician guidance and factors they consider important in value-laden conversations with healthcare professionals.

## Methods

### Study design

This qualitative study represents a secondary analysis embedded within a previously reported web-based randomized controlled trial (RCT) [12]. The main trial investigated whether disclosure of a clinician’s personal opinion influences parental decision-making in a simulated neonatal scenario.

Participants were presented with an online video-based vignette describing a critically ill female infant born extremely prematurely at 23 weeks of gestation with a birth weight of 530 g. The infant required prolonged invasive ventilation and had been diagnosed with severe bilateral intracranial hemorrhage and seizures. At four months of age, she remained in intensive care with invasive ventilation, and the possibility of performing a tracheostomy was discussed with her parents. During this conversation, the mother asked the physician: “What would you do if it were your child?”.

Two versions of the physician’s response were developed: one including disclosure of the clinician’s personal opinion (intervention) and one without such disclosure (control). The responses were structured using the “ANSWER” mnemonic (Active listening, Needs assessment, Self-awareness, Whose values, Elicit values, Respond) [13]. The scenario was presented using animated video sequences.

After viewing the scenario, participants provided free-text responses to an open-ended question regarding factors they considered important in conversations with clinicians about treatment decisions for their child. The results of the RCT have been reported elsewhere [12].

This qualitative study was approved by the Ethics Commission of the Canton of Berne (Req-2023-00884), Switzerland, which declared that the study did not fall under the provisions of Article 2, Paragraph 1 of the Swiss Federal Law on Human Research. All participants provided electronic informed consent prior to enrolment. This study followed the Standards for Reporting Qualitative Research (SRQR) reporting guideline [14].

### Participants and recruitment

Participants were German-speaking parents (≥ 18 years) of preterm born infants who had received neonatal intensive care. Recruitment occurred between February and April 2024 via social media and newsletters from the parent and patient organizations “Frühchen & Neokinder Schweiz” in Switzerland and “Bundesverband das Frühgeborene Kind” in Germany and Austria. Participants were randomized 1:1 using the German SoSciSurvey platform.

### Analysis

A qualitative content analysis was conducted following the approach described by Mayring [15]. The analysis was primarily inductive, allowing categories to emerge from the data.

Parental free-text responses to the question “What factors are important to you in conversations with clinicians regarding treatment decisions for your child?” were analyzed. Two investigators (JE and CA) independently familiarized themselves with the data through repeated reading. Initial codes were generated through line-by-line coding and subsequently refined and grouped into broader categories. Through an iterative process of comparison and discussion, categories and subcategories were refined. Discrepancies were resolved through discussion until consensus was reached. Representative anchor quotes were selected to illustrate categories and translated into English. In the last step, by identifying patterns, relationships, and underlying concepts within the data we created main categories. The research team included individuals with different levels of experience in qualitative research. CA had experience in qualitative methods, neonatal care as a nurse, and health services research, while JE was a medical student without prior experience in qualitative research. Regular discussions and supervision supported the analytical process and enhanced reflexivity.

Finally, we examined the qualitative data for potential differences across sociodemographic subgroups, including gender, place of birth, university degree, and whether the infant was discharged before or after 14 days. The qualitative responses were reviewed from the perspective of clinical guidance to identify subgroup-specific patterns. To complement this qualitative exploration, we conducted a descriptive analysis of participants’ responses to the question: “How helpful do you think it is when physicians disclose their personal opinions in order to make a treatment decision in such a situation?” Mean scores and standard deviations were calculated for each subgroup. Responses were rated on a 10-point Likert scale ranging from 1 (not very helpful) to 10 (very helpful).

## Results

In total, 218 participants were included in the RCT, of whom 128 provided free-text responses on factors they considered important in conversations with clinicians regarding treatment decisions for their child.

Demographic characteristics are summarized in Table 1. The study sample included 108 (84.4%) female participants, and 67 (52.3%) had no university degree. The mean (SD) age of participants was 36.7 (5.3) years. The gestational age of participants’ infants ranged from 23.0 to 36.7 weeks, with a mean (SD) of 29.7 (3.6) weeks. These demographic characteristics were comparable to those reported in the RCT [12].

**Table 1:** Sociodemographic characteristics

Characteristics	*N* (%) *N* = 128
**Parents**	
Gender	
Female	108 (84.4)
Male	20 (15.6)
Age in years, mean (SD) [range]; *n* = 127	36.7 (5.3); [24–57]
Place of birth	
Switzerland	66 (51.6)
Germany	51 (39.8)
Austria	6 (4.7)
Other	4 (3.1)
Not answered	1 (0.8)
University degree	
No	67 (52.3)
Yes	60 (46.9)
Not answered	1 (0.8)
**Infants**	
Gestational age in weeks, mean (SD) [range]	29.7 (3.6); [23.0–36.7]
Place of birth	
Switzerland	75 (58.6)
Germany	42 (32.8)
Austria	6 (4.7)
Other	1 (0.8)
Not answered	4 (3.1)
Discharge after	
≤ 14 days	15 (11.7)
> 14 days	108 (84.4)
Deceased	2 (1.6)
Not answered	3 (2.3)
Respiratory support at discharge	
No breathing support	109 (85.2)
Supplemental oxygen	9 (7.0)
Non-invasive ventilation (CPAP or high flow)	4 (3.1)
Tracheostomy tube	3 (2.3)
Not answered	3 (2.3)

*Abbreviations*: *SD* Standard deviation, *CPAP* Continuous Positive Airway Pressure

The factors identified by participants as important in conversations with clinicians regarding value-laden treatment decisions for their child were grouped into eight categories: evidence-based medicine, comprehensive information (including benefits and risks), experiences and personal opinion, empathetic communication, honesty, personalized communication, time, and support. In addition, some participants reflected on the specific case presented in the video animations, highlighting the burden of decision-making, considerations of the child’s best interests, and the importance of quality of life. The responses ranged from single words to short paragraphs of about 100 words. The identified categories and subcategories, along with representative anchor quotes, are presented in Table 2.

**Table 2:** Important factors in conversations with clinicians about treatment decisions

Categories	Subcategories	Anchor quotes
Evidence-based-medicine	• Medical factors • Expertise • Competence • Statistics • Objectivity • Facts	*“What does the medical condition specifically mean?”* *“Expert knowledge. It is mentally exhausting for a mother to have to read guidelines and research how to help her child.”*
Comprehensive information, including the benefits and risks	• Comprehensive information • Provide clarifications and list alternatives • Pros and cons • Be aware of all options • Risks and consequences	*“To feel completely informed about all options*,* consequences*,* side effects*,* etc.”* *“To be educated about all the possibilities along with their respective risks and consequences.”*
Experiences and personal opinion	• Pros and cons personal opinion • Experiences • Personal assessment • Recommendations	*“Would the physician subject her own child to this treatment*,* or is it too burdensome and does not lead to healing?”* *“I prefer personal assessments from physicians*,* as I can make a better decision for or against something based on direct statements rather than vague ones.”*
Empathetic communication	• Empathy • Compassion • Understanding • Trust • Sympathy • Conversation at eye level • The ability to ask questions • Calm • Acceptance of the parents’ opinion • Optimism	*“The doctor must be kind and empathetic. He or she should not be too cold-hearted*,* yet should present all the facts*,* as long as they believe this will not further traumatize the parents.”* *“Compassion*,* but still radiating confidence*,* so that I can trust the physician.”*
Honesty	• Honesty • Openness • Transparency	*“Honesty*,* not beating around the bush*,* but clearly stating how the situation is!”* *“Everything is built upon an honest conversation*,* and everything else follows from that.”*
Personalized communication	• Individual assessment of the situation • Involving the parents	*“The physician should adapt the conversation based on the parents and not just follow a set script.”*
Time	• Time for questions • Consideration time • Reflection	*“It would be very important to me that*,* after such a pivotal conversation*,* I can discuss what was said privately with my wife*,* and also take the time to think about it and reflect on it by myself.”*
Support	• Support options • Involving additional healthcare professionals	*“What support options are available? To be accompanied and not left alone (no one feels responsible).”*

Parents consistently emphasized the importance of receiving clear, evidence-based information. Many sought factual explanations and, where possible, statistical data to better understand their child’s condition and likely disease trajectory. They also expected clinicians to provide clear and detailed interpretations of what the clinical picture meant for their individual child. Beyond factual information, parents highlighted the need for comprehensive discussions of treatment options, including all available alternatives and their associated risks, benefits, and long-term consequences. Participants emphasized the importance of transparent explanations of advantages and disadvantages to support informed deliberation.

A central factor concerned clinicians’ personal opinions and recommendations. Some parents expressed a preference for clinicians to share their personal assessments, viewing this as a form of professional engagement and guidance in complex decision-making situations. They suggested that clear and direct statements could facilitate weighing options and support decision-making, particularly in situations of uncertainty. In contrast, other parents expressed reservations about clinicians disclosing personal opinions. They noted that such views may be shaped by individual experiences and values and could influence how treatment options are presented. These participants preferred a more neutral and balanced presentation of information, allowing them to consider options in light of their own priorities. Overall, responses reflected ambivalence regarding the role of clinician guidance, with differing preferences for explicit recommendations versus value-sensitive neutrality. Across these perspectives, parents appeared to retain responsibility for decision-making, as reflected in their emphasis on understanding options, taking time for reflection, and considering decisions in light of their own values, while differing in how much guidance they wished to receive from clinicians.

Time was identified as an important factor in several respects. Parents emphasized the need for sufficient time during discussions, as well as time afterward to reflect, discuss options with others, and seek additional information. Relational aspects of communication were also central. Parents highlighted the importance of empathetic communication characterized by compassion, sensitivity, and respect. They valued conversations conducted on equal footing, with opportunities to ask questions and express their views. In addition to empathy, honesty, openness, and transparency were considered essential, alongside a desire for realistic optimism. Parents also emphasized the importance of support during decision-making processes. Many mentioned the value of second opinions and input from additional healthcare professionals. A recurring concern was the desire not to feel abandoned, with some participants noting a lack of clear responsibility in challenging situations.

Finally, participants stressed the importance of individualized communication. Physicians were expected to adapt their approach to the specific needs and circumstances of each family rather than following a standardized script. Consideration of the child’s quality of life was also frequently highlighted, particularly in relation to potential suffering associated with different treatment options.

**Fig. 1:**
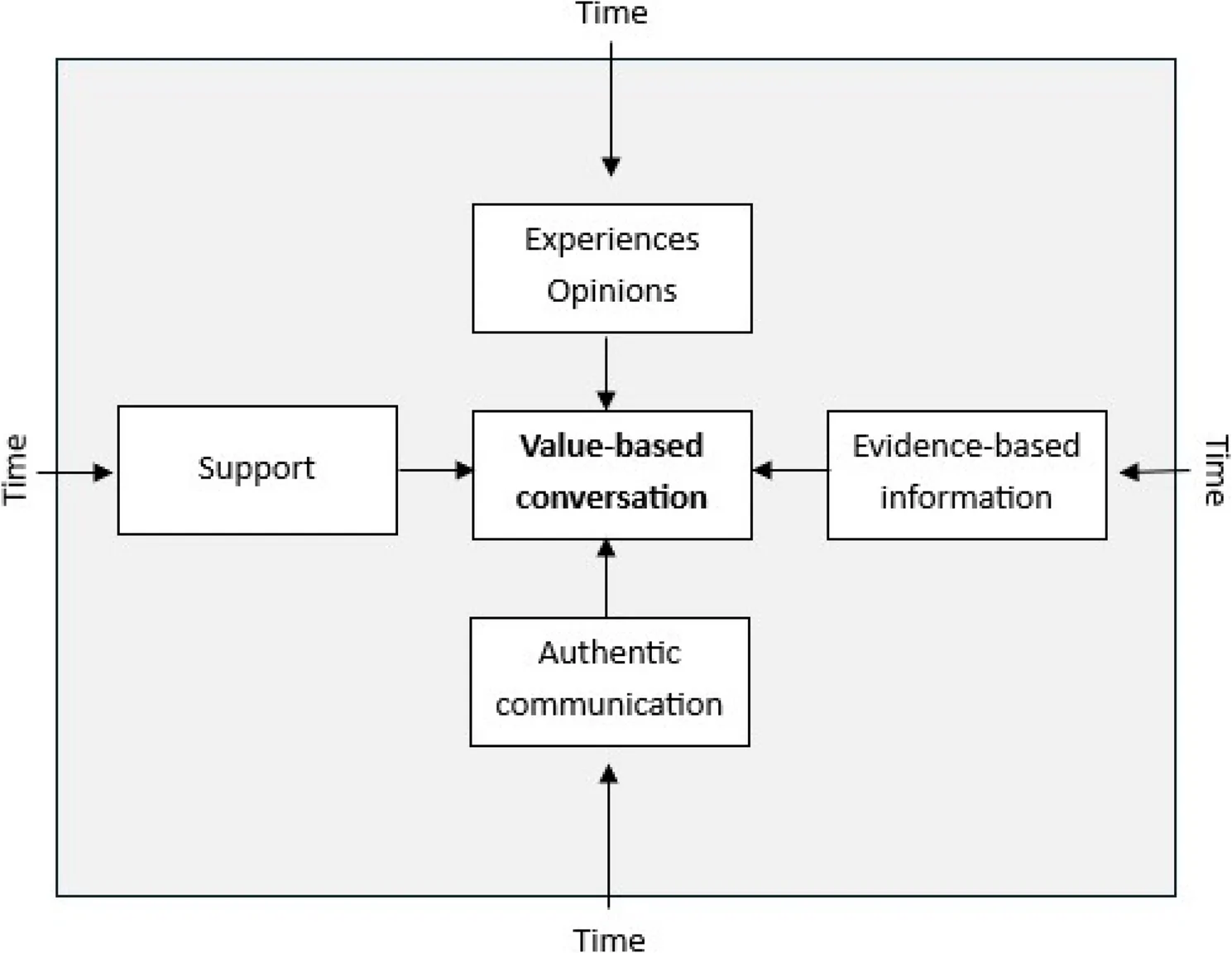
Main categories

Through iterative refinement of the coding system in team discussions, five main categories were identified that capture key factors influencing value-based conversations with parents (see Fig. 1). The categories empathic communication, honesty, and personalized communication were subsumed under the main category authentic communication. Likewise, evidence-based-medicine and comprehensive information were merged into the main category evidence-based information. The categories support and experiences and personal opinion remained distinct. Time emerged as a cross-cutting factor affecting all categories and was therefore conceptualized as an overarching contextual condition that shapes value-based conversations.

Regarding potential differences across participant subgroups (e.g., according to sociodemographic characteristics), the qualitative categories and overall sentiments were highly consistent, with no distinct subgroup-specific patterns or unique sentiments emerging from the data. Table 3 presents the mean assessment scores for clinicians’ disclosure of personal opinions in value-laden NICU conversations according to participants’ sociodemographic characteristics. No relevant differences were observed between the sociodemographic groups.

**Table 3:** Mean assessment of clinicians’ personal opinion disclosure by participants’ sociodemographic characteristics

Characteristics	Helpfulness of physician opinion disclosure Mean (SD)
Gender	
Female	6.4 (2.5)
Male	6.5 (2.4)
Place of birth	
Switzerland	6.7 (2.3)
Other country	6.2 (2.7)
University degree	
No	6.5 (2.5)
Yes	6.2 (2.5)
Infants discharge after	
≤ 14 days	6.1 (2.7)
> 14 days	6.4 (2.5)

*Abbreviations*: *SD* Standard deviation

## Discussion

In this qualitative study, parents expressed differing expectations regarding clinicians’ personal recommendations in value-laden NICU conversations. While some welcomed explicit guidance, others preferred a more neutral approach that allowed decisions to be shaped primarily by their own values and priorities. Across these perspectives, parents appeared to retain responsibility for decision-making, while differing in how much guidance they wished to receive from clinicians. These findings suggest that decision-making in this context is best understood as a supported process in which parents retain responsibility while drawing on professional guidance to navigate uncertainty and emotional burden. Rather than representing a transfer of responsibility, clinician input appears to function as a resource that parents may engage with to varying degrees.

Study results align with previous research highlighting the importance of shared decision-making in neonatal care, where parents act as surrogate decision-makers for their children. Shared decision-making aims to integrate clinical expertise with the values and preferences of families [6, 16, 17]. Our findings extend this perspective by highlighting that parents differ not only in their needs, but also in how much clinician guidance they prefer within this process.

The ambivalence regarding clinicians’ personal opinions reflects an ongoing challenge in neonatal care. While some parents experienced recommendations as supportive and clarifying, others were concerned about potential bias and preferred a more balanced presentation of options. This variation underscores that no single approach to clinician guidance is appropriate for all parents. Parental expectations toward clinicians therefore appear to be dynamic and context-dependent. They may vary according to the clinical situation, perceived severity, and individual coping strategies. Future research could further investigate whether parental preferences regarding clinician communication vary according to these factors.

Clinicians may need to adapt their communication style by explicitly exploring parents’ preferences regarding professional recommendations rather than assuming a uniform approach. In this sense, shared decision-making involves not only the exchange of information but also an explicit and repeated negotiation of how actively clinicians should contribute their professional judgment.

Our findings also emphasize that communication in these situations is inherently relational. Trust, emotional support, and mutual understanding appear to be central for enabling parents to engage in complex decision-making. This is consistent with principles of family-centered and family integrated care, which recognize parents as essential partners in the care of their child [11, 18, 19].

Overall, these findings highlight that value-laden NICU conversations require not only the provision of information but also careful attention to how guidance is offered and how parents wish to engage in the decision-making process. Clinicians therefore need skills to support parents in reflecting on their own values and to empower them to make informed decisions. Training opportunities are needed to practice these conversations, and their effectiveness should be evaluated in randomized controlled trials [20, 21].

### Strengths and limitations

A strength of this study is the inclusion of perspectives from more than 100 parents from German-speaking countries regarding important aspects of value-laden conversations. Our study is one of the few to focus on clinician guidance in value-laden NICU conversations. However, several limitations should be considered. First, parents responded to a simulated clinical scenario presented through video animations rather than an actual decision-making situation, which may not fully capture the emotional complexity of real-life contexts. Nevertheless, the study’s findings were consistent with the existing literature, which strengthens confidence in their validity. This concordance suggests that, despite the simulated format, parents were able to meaningfully engage with the scenario and provide responses that approximate real-world perspectives.

Second, participants were recruited through parent organizations and social media channels, which may have attracted parents who are particularly engaged or interested in neonatal care. Their perspectives may therefore not fully represent the experiences of all parents of preterm infants. In addition, mothers were overrepresented in the sample, which is common in parental research but may limit insights into fathers’ perspectives. In addition, culturally and linguistically homogeneous population may limit generalizability. Third, another potential selection bias must be mentioned because only 59% participants of the RCT response of the open-ended questions about influence factors in value-based communication which is analyzed in this study. The length of the video animations from the RCT may have contributed to participant dropout.

## Conclusions

These findings underscore that effective communication in value-laden NICU conversations involves more than the transmission of medical facts. While parents appear to retain responsibility for decision-making, preferences regarding clinician guidance vary, highlighting the importance of tailoring communication and explicitly exploring how much direction parents wish to receive.

## Data Availability

The datasets used and/or analyzed during the current study are available from the corresponding author on reasonable request.

## Abbreviations

NICU: Neonatal intensive care unit
RCT: Randomized controlled trial
SD: Standard deviation
